# Association between blood heavy metal levels and COPD risk: a cross-sectional study based on NHANES data

**DOI:** 10.3389/fpubh.2025.1494336

**Published:** 2025-07-18

**Authors:** Xingshi Hua, Lingyun Zhu, He Chen

**Affiliations:** ^1^Liaoning University of Traditional Chinese Medicine, Shenyang, China; ^2^Liaoning University of Traditional Chinese Medicine Affiliated Second Hospital, Shenyang, China; ^3^The First Affiliated Hospital of Liaoning University of Traditional Chinese Medicine, Shenyang, China

**Keywords:** chronic obstructive pulmonary disease, heavy metals, cross-sectional study, National Health and Nutrition Examination Survey, receiver operating characteristic analysis, environmental exposure assessment

## Abstract

**Objective:**

Heavy metals such as cadmium (Cd), lead (Pb), mercury (Hg), selenium (Se), and manganese (Mn) are commonly found in the environment and could increase the risk of COPD, potentially through key biological mechanisms such as oxidative stress and inflammation. Nonetheless, evidence on the association between exposure to these metals and COPD remains limited and inconclusive. This study aims to evaluate the potential association between blood levels of Cd, Pb, Hg, Se, and Mn and the likelihood of developing COPD.

**Methods:**

This cross-sectional analysis utilized data from 7,458 individuals from the NHANES. COPD status was determined based on self-reported physician diagnoses obtained from questionnaires. Multivariate logistic regression models were applied to examine the association between blood heavy metal levels and the risk of COPD. Subgroup analyses were conducted to explore potential differences in these associations across various population groups. Additionally, smoothing curve analysis was used to investigate nonlinear relationships between heavy metal concentrations and COPD. The receiver operating characteristic (ROC) curve analysis was employed to assess the predictive value of blood metal levels for COPD risk.

**Results:**

Blood levels of Cd and Pb were markedly elevated in COPD patients compared to those without the condition. Even after controlling for multiple confounders, higher blood levels of Cd and Pb were found to be significantly associated with a greater risk of developing COPD [odds ratio (OR) = 1.27, 95% confidence interval (CI): 1.13–1.44 and OR = 1.69, CI: 1.13–1.50, respectively; both *p* < 0.001]. The smoothing curve analysis demonstrated a nonlinear positive relationship between Cd and Mn levels and the risk of COPD. ROC curve analysis showed that Pb had the highest Area Under the Curve (AUC) value of 0.6744, suggesting different levels of sensitivity and specificity for these heavy metals in predicting COPD risk.

**Conclusion:**

This study highlights that a significant association was observed between blood levels of Cd and Pb and the likelihood of developing COPD, with a significant trend across exposure quartiles. Moreover, the data suggest a nonlinear positive correlation between exposure to Cd and Mn and COPD. ROC analysis further revealed differential sensitivity and specificity among the five heavy metals in predicting COPD risk.

## 1 Introduction

Chronic obstructive pulmonary disease (COPD) is a growing public health concern characterized by irreversible airflow limitation that typically worsens over time (1). As a major chronic disease, COPD poses a significant threat to human health. COPD is the third leading cause of death globally, with its prevalence rising due to aging populations and continued exposure to risk factors such as tobacco smoke and air pollution. It is now a key focus of global chronic disease prevention efforts (2–5). Epidemiological studies indicate that individuals over the age of 40 are at high risk for COPD, with a global prevalence of approximately 9–10% in this age group (6). In recent years, the number of COPD patients in China has been increasing, driven by the aging population. In line with this trend, a systematic analysis of the 2019 Global Burden of Disease Study reported that approximately 1.04 million deaths in China were attributable to COPD in that year (7). A nationwide cross-sectional survey reported a COPD prevalence of 8.6% among individuals aged 20 and above in China (8). In rural areas, COPD has long been the leading cause of death, while in urban settings, it ranks fourth. With an aging population, rising smoking rates, and persistent air pollution, the incidence and mortality of COPD in China continue to increase annually, posing significant challenges to healthcare systems and economic sustainability (9, 10). Given the incurable nature of COPD, understanding its etiology and pathogenesis, preventing its onset and progression, and mitigating further deterioration are critical research priorities.

In recent years, environmental pollution, particularly heavy metal contamination, has emerged as a severe global environmental health issue. In environmental health, heavy metals refer to metals with significant biological toxicity, such as cadmium (Cd) and lead (Pb) (11). Pollution caused by heavy metals and their compounds is termed heavy metal pollution. This type of pollution represents a worldwide environmental challenge, causing substantial harm to human health and socioeconomic stability. Studies suggest that environmental factors contribute to 24% of the global disease burden, with 17% of deaths in developed regions and 25% in developing regions attributable to these factors (11). Among these environmental pollutants, heavy metals pose a significant threat, particularly to the respiratory system, as inhalation is a primary exposure route (12). In developing countries and underdeveloped regions, health losses due to heavy metal pollution, measured in disability-adjusted life years (DALYs), have reached 14.75 million (13). Globally, approximately 20 million hectares of arable land are affected by metal contamination, accounting for 16% of the world’s total arable land and covering 10 million terrestrial sites, leading to an estimated annual economic loss of $10 billion (14).

Heavy metals pose long-term threats to human health due to their toxicity, environmental persistence, and bioaccumulation properties. These metals are widely present in the atmosphere, water, and soil, primarily originating from industrial emissions, mining, smelting, coal combustion, vehicle exhaust, wastewater discharge, and the use of fertilizers and pesticides in agriculture. Heavy metals in the environment can adversely affect health not only through direct exposure but also by contributing to air pollution, which negatively impacts the respiratory system. Studies have shown that both indoor and outdoor air pollution increase the incidence of COPD, the frequency of acute exacerbations, hospitalization rates, and mortality (15).

Cd, a toxic heavy metal, is widely used in the production of mineral fertilizers, plastics, batteries, pigments, and metal coatings. In humans, the primary routes of Cd exposure include ingestion of polluted food and water, inhalation of cigarette smoke, and breathing in airborne particulate matter (PM2.5) (16). Cigarette smoke contains significant amounts of Cd, and Cd oxide formed during smoking can either accumulate in pulmonary tissues or be taken up into the bloodstream (17). Cd also accumulates in soil and can be taken up by crops such as fruits, vegetables, and rice, making diet a major source of Cd exposure for non-smokers (18). Additionally, occupational exposure to Cd dust has been linked to an increased risk of COPD. Research indicates that Cd inhalation is more efficiently absorbed into the lungs than through the gastrointestinal tract (19). After entering the human system, Cd binds to red blood cells and albumin, subsequently building up in organs such as the liver and kidneys. With a biological half-life spanning 20 to 30 years, this accumulation leads to significant and prolonged health hazards (20).

In addition to Cd, exposure to heavy metals like Pb and mercury (Hg) has also been linked to an increased risk of developing COPD. Pb can exacerbate chronic lung inflammation and cause tissue damage by suppressing antioxidant enzyme activity and enhancing the generation of reactive oxygen species (ROS) (21). Hg disrupts cellular redox balance and induces immune dysregulation, affecting the progression of COPD (22). While selenium (Se) and manganese (Mn) are essential trace elements at normal levels, excessive exposure can lead to oxidative stress and inflammatory responses, exacerbating respiratory damage (23).

Despite growing evidence linking heavy metal exposure to respiratory health, the relationship between specific heavy metals and COPD remains inadequately explored. The plausibility of such an association is supported by toxicological evidence demonstrating that heavy metals can induce pulmonary inflammation and oxidative stress, which are core mechanisms in the pathogenesis of COPD. While previous research has suggested potential associations between certain heavy metals and respiratory diseases, comprehensive studies on co-exposure to multiple metals in large, representative populations remain limited (24). Additionally, the predictive capabilities of these metals for COPD risk assessment remain largely unexplored.

This study investigates the associations between five specific heavy metals—Cd, Pb, Hg, Se, and Mn—and COPD using data from the NHANES 2017–2020 dataset. We specifically selected this most recent NHANES cycle as it provides the most up-to-date nationally representative data on heavy metal exposure and respiratory health in the United States, allowing for contemporary insights into these relationships. Our research advances current knowledge by simultaneously assessing multiple heavy metals within a nationally representative cohort, applying advanced statistical methods including nonlinear smoothing curve analysis to detect potential dose–response relationships, and evaluating each metal’s sensitivity and specificity in predicting COPD risk through ROC analysis. These methodological approaches collectively enable a more comprehensive understanding of how different heavy metals might contribute to COPD development. We hypothesize that elevated exposure to these heavy metals correlates with increased COPD risk.

## 2 Methods

### 2.1 Study design and sample

This research analyzed data from the National Health and Nutrition Examination Survey (NHANES) spanning 2017 to 2020, a period for which the 2019–2020 data collection was incomplete due to the COVID-19 pandemic, to assess the association between exposure to heavy metals and the prevalence of COPD. NHANES, conducted across the United States, employs a multistage, stratified sampling approach to gather extensive information on the health, nutrition, and demographics of individuals nationwide. The intricate sampling strategy ensures that the results are applicable to the entire U.S. population. Informed consent was obtained from all individuals involved, and the study protocol received approval from the Ethics Review Board at the National Center for Health Statistics (NCHS). Additional details about the survey are available on the Centers for Disease Control and Prevention (CDC)'s official website.^1^

**Figure 1:**
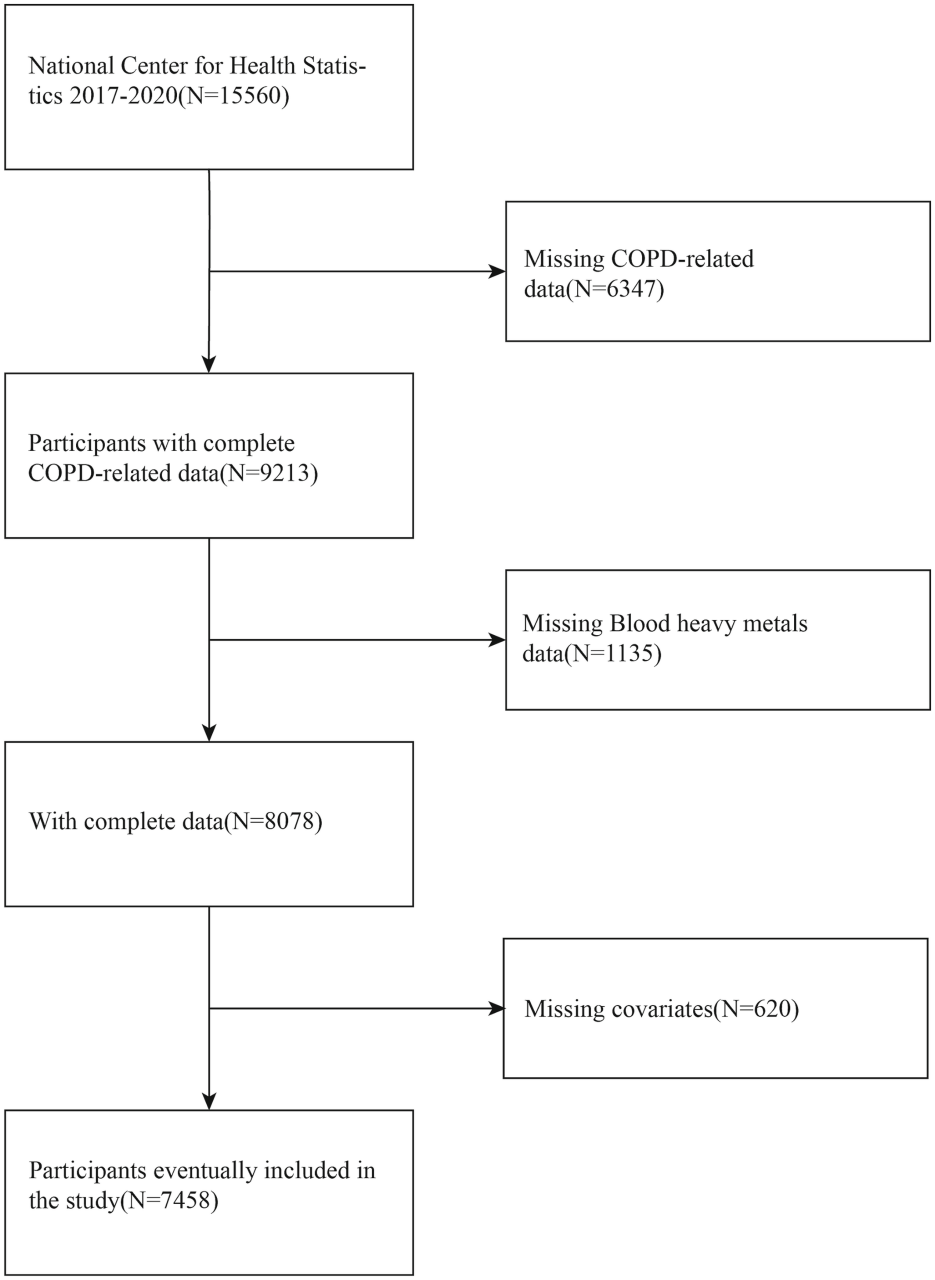
Sample flowchart. COPD, Chronic obstructive pulmonary disease.

The preliminary analysis comprised 7,458 qualified participants. The criteria for exclusion were defined as follows: (1) individuals without COPD diagnostic data, (2) individuals lacking heavy metal exposure data, and (3) individuals missing data on covariates. This approach constitutes a complete case analysis, where only participants with full data on all variables of interest were included in the final models. These criteria were applied to ensure data completeness and the accuracy of the analysis. Given that COPD is predominantly a disease of advanced age, the potential for confounding by pregnancy was considered negligible; thus, it was not used as an explicit exclusion criterion. Medical comorbidities, such as asthma, diabetes, and heart disease, were also not excluded but were instead adjusted for as potential confounders to maintain the external validity of our findings. A comprehensive flowchart detailing the selection process of the study population is shown in Figure 1.

NHANES data collection encompasses various data types, including demographic information, dietary records, physical examination results, laboratory test data, and health questionnaires. All study procedures underwent rigorous ethical review and were conducted with informed consent from participants. Data from the 2017 to 2020 NHANES cycle were selected for analysis due to the recency and comprehensive measurement of heavy metal levels during this period. A total of 7,458 participants meeting all inclusion criteria were analyzed.

### 2.2 Exposure variable: heavy metal levels

In this study, the assessment of heavy metal exposure levels was conducted by analyzing whole blood samples from participants. Blood levels of Pb, Cd, Hg, Mn, and Se were quantified using Inductively Coupled Plasma-Mass Spectrometry (ICP-MS). Detailed laboratory procedures and documentation are publicly available on the NHANES website (25). Mass spectrometry is an analytical method known for its high sensitivity and specificity, enabling accurate detection and measurement of various metal elements in a sample. Prior to measurement, the samples underwent a simple dilution step to reduce complexity and enhance analytical accuracy. This method allowed for the direct measurement of heavy metal concentrations in participants, thereby offering a more precise assessment of the potential link between heavy metal exposure and COPD risk. The concentrations of all five metals (Pb, Cd, Hg, Se, and Mn) were reported in μg/L.

### 2.3 Outcome variable: COPD

In this study, the diagnosis of COPD was determined through self-reported data collected via questionnaires. The key survey questions asked participants whether they had ever been diagnosed with COPD, emphysema, or chronic bronchitis by a doctor or other healthcare professional. This strategy successfully pinpointed individuals diagnosed with COPD, offering essential data for examining the link between COPD and exposure to heavy metals.

### 2.4 Covariates

This study utilized multivariable adjustment models to control for potential confounders that might affect the relationship between heavy metal concentrations and COPD. The covariates included in the analysis encompassed a range of demographic and health-related factors that might affect the relationship between exposure and outcome. The adjustments in the analysis specifically included covariates such as age, gender, ethnicity, smoking habits, alcohol intake, body mass index (BMI), and medical history, including conditions like cancer and asthma. Information on demographic characteristics, health behaviors (smoking, alcohol), and medical history was collected via standardized questionnaires. BMI was calculated from height and weight measured during the physical examination. The selection and measurement of these variables were guided by their relevance in previous studies and the objectives of the current study to ensure analytical accuracy and scientific rigor (24, 26, 27).

To better control for confounding bias, a systematic approach was employed to identify and handle covariates. It is important to note that not all covariates are considered confounders; only those variables that are significantly associated with both heavy metal exposure and COPD risk were treated as confounding factors requiring adjustment. Table 1 provides detailed definitions and measurement methods for each covariate. These variables were selected based on evidence from prior studies, aiming to reduce potential confounders and better elucidate the association between heavy metal concentrations and COPD. Details on measurement techniques and data sources can be found on the CDC website. Adjusting for these covariates allows for a more precise evaluation of the independent effects of heavy metal exposure on COPD risk, thereby mitigating the influence of confounders and offering stronger epidemiological insights.

**Table 1:** Baseline characteristics of participants in the NHANES 2017–2020, categorized by COPD status.

Characteristic	Non-COPD	COPD	*p*-value
	*N* = 6,782 (90.9%)	*N* = 676 (9.1%)	
Age (year)	49.92 ± 17.32	60.37 ± 14.99	<0.001
BMI (kg/m^2^)	29.92 ± 7.28	31.69 ± 9.32	<0.001
Pb (μg/L)	1.17 ± 1.22	1.38 ± 1.11	<0.001
Cd (μg/L)	0.44 ± 0.50	0.77 ± 0.81	<0.001
Hg (μg/L)	1.36 ± 2.33	1.00 ± 1.67	<0.001
Se (μg/L)	186.01 ± 26.96	183.86 ± 31.04	0.007
Mn (μg/L)	9.73 ± 3.62	9.51 ± 3.53	0.045
Gender (%)			0.541
Male	3,324 (49.01%)	323 (47.78%)	
Female	3,458 (50.99%)	353 (52.22%)	
Race (%)			<0.001
Mexican American	857 (12.64%)	27 (3.99%)	
Other Hispanic	720 (10.62%)	50 (7.40%)	
Non-Hispanic White	2,324 (34.27%)	380 (56.21%)	
Non-Hispanic Black	1,763 (26.00%)	153 (22.63%)	
Non-Hispanic Asian	815 (12.02%)	18 (2.66%)	
Other Race—Including Multi-Racial	303 (4.47%)	48 (7.10%)	
Education level (%)			<0.001
Less than 9th grade	477 (7.03%)	40 (5.92%)	
9–11th grade	691 (10.19%)	105 (15.53%)	
High school graduate	1,602 (23.62%)	211 (31.21%)	
Some college or AA degree	2,237 (32.98%)	248 (36.69%)	
College graduate or above	1,775 (26.17%)	72 (10.65%)	
Marital status (%)			<0.001
Married	4,014 (59.19%)	332 (49.11%)	
Single	2,768 (40.81%)	344 (50.89%)	
Alcohol (%)			<0.001
Drinks	6,151 (90.70%)	641 (94.82%)	
Never	631 (9.30%)	35 (5.18%)	
Hypertension (%)			<0.001
Yes	2,476 (36.51%)	390 (57.69%)	
No	4,306 (63.49%)	286 (42.31%)	
Diabetes (%)			<0.001
Yes	1,141 (16.82%)	217 (32.10%)	
No	5,641 (83.18%)	459 (67.90%)	
Asthma (%)			<0.001
Yes	900 (13.27%)	289 (42.75%)	
No	5,882 (86.73%)	387 (57.25%)	
Heart attack (%)			<0.001
Yes	235 (3.47%)	102 (15.09%)	
No	6,547 (96.53%)	574 (84.91%)	
Cancer (%)			<0.001
Yes	660 (9.73%)	132 (19.53%)	
No	6,122 (90.27%)	544 (80.47%)	
Smoking status (%)			<0.001
Yes	2,659 (39.21%)	497 (73.52%)	
No	4,123 (60.79%)	179 (26.48%)	

### 2.5 Statistical analyses

To maintain national representativeness and enhance statistical reliability, this study incorporated NHANES’ complex sampling framework and sample weighting. The statistical analyses were carried out with R software version 4.0.5 and EmpowerStats version 2.0, applying a threshold of *p* < 0.05 for significance in all tests. To summarize the main characteristics of the study population, descriptive statistics were initially employed. Categorical data were expressed as weighted percentages, while continuous data were summarized using weighted means and standard deviations. The Chi-square test was utilized to compare categorical variables, and analysis of variance (ANOVA) was used to examine differences among continuous variables. This step provided a foundational understanding of the baseline characteristics and differences among different groups, setting the stage for subsequent analyses.

Three multivariable logistic regression models were developed to examine the association between concentrations of heavy metals and the risk of COPD. Model I served as a baseline model without any covariate adjustment, providing an unadjusted estimate of the association. Model II adjusted for race, sex, and age, controlling for basic demographic factors. Model III included further adjustments for other confounders such as marital status, educational attainment, BMI, alcohol use, smoking habits, hypertension, diabetes, asthma, heart disease, and cancer. This incremental adjustment approach enabled a systematic evaluation of how each covariate influenced the link between exposure to heavy metals and COPD. The odds ratio (OR) and its 95% confidence interval (CI) were computed to better represent the independent effect of heavy metal exposure on the risk of developing COPD.

Furthermore, we performed subgroup analyses and interaction tests to determine if the relationships between heavy metal exposure and COPD varied among different population groups, specifically examining Pb and Cd. This approach helped identify potentially susceptible groups or differential effects under specific conditions.

To investigate possible nonlinear relationships between heavy metal exposure and the risk of COPD, the study utilized smoothing curve fitting methods to examine the associations of Pb and Mn with COPD. This method allowed for the identification of complex dose–response patterns between exposure levels and outcomes.

Finally, to assess the predictive effectiveness of each heavy metal indicator for COPD risk, receiver operating characteristic (ROC) curve analysis and measurements of the area under the curve (AUC) were conducted. ROC analysis helped determine optimal cutoff points for prediction and evaluated the sensitivity and specificity of each heavy metal as a potential marker for COPD, providing evidence to guide public health interventions.

## 3 Results

### 3.1 Baseline characteristics of participants

The weighted demographic data and covariates of participants, categorized by COPD status, are summarized in Table 1. Out of the 7,458 individuals included in the study, 676 were identified as having COPD. Within the COPD group, 47.78% were male. The weighted mean age of COPD patients was 60.37 ± 14.99 years, which is significantly older compared to 49.92 ± 17.32 years for participants without COPD. Furthermore, the weighted mean body mass index (BMI) for COPD patients was 31.69 ± 9.32, which was slightly higher than the BMI of 29.92 ± 7.28 for non-COPD participants. The prevalence of alcohol consumption, hypertension, diabetes, asthma, a history of heart attack, cancer, smoking, and being single was significantly higher among COPD patients compared to non-COPD participants (all *p* < 0.001, as determined by chi-square tests for categorical variables and ANOVA for continuous variables). Nevertheless, the gender distribution did not differ significantly between the groups (*p* = 0.541). It is noteworthy that blood levels of Pb and Cd were markedly elevated in individuals with COPD compared to those without, with these differences reaching statistical significance (*p* < 0.001).

### 3.2 Association between heavy metal levels and COPD risk

Upon adjusting for all pertinent covariates, the analysis demonstrated that for each unit increase in blood Cd levels, the risk of COPD rose by 105% (OR = 2.05, 95% CI: 1.79–2.34, *p* < 0.001). This indicates that higher blood Cd levels are significantly linked to an elevated risk of developing COPD. Further stratification by quartiles of blood Cd revealed that participants in the highest quartile (Q4) faced a 317% greater risk of COPD compared to those in the lowest quartile (Q1) (OR = 4.17, 95% CI: 3.12–5.58, *P* for trend < 0.01) (Table 2).

**Table 2:** Multivariable logistic regression models for the association between blood Cd and COPD.

Variable	Model I (unadjusted)		Model II (demographics adjusted)		Model III (fully adjusted)	
	OR (95% CI)	*p*-value	OR (95% CI)	*p*-value	OR (95% CI)	*p*-value
Cd Continuous	2.06 (1.85, 2.30)	<0.0001	2.08 (1.85, 2.34)	<0.0001	2.05 (1.79, 2.34)	<0.0001
Categories						
Quartile1	Ref		Ref		Ref	
Quartile2	1.27 (0.94, 1.70)	0.1223	1.00 (0.74, 1.36)	0.9818	1.03 (0.75, 1.42)	0.8596
Quartile3	2.12 (1.61, 2.79)	<0.0001	1.56 (1.17, 2.08)	0.0024	1.73 (1.27, 2.35)	0.0005
Quartile4	4.54 (3.52, 5.85)	<0.0001	3.82 (2.93, 4.97)	<0.0001	4.17 (3.12, 5.58)	<0.0001
*P* for trend	6.73 (5.21, 8.69)	<0.0001	6.64 (5.06, 8.71)	<0.0001	7.19 (5.29, 9.76)	<0.0001

OR, odds ratios; CI, confidence intervals; Ref, Reference. Model I: Unadjusted, without covariate adjustment; Model II: Adjusted for basic demographic factors, including age, sex, and race; Model III: Fully adjusted for demographic, behavioral, and health-related factors, including marital status, educational attainment, BMI, smoking habits, alcohol use, and comorbidities such as hypertension, diabetes, asthma, heart disease, and cancer.

In a similar vein, analysis of blood Pb concentrations indicated that, after accounting for all possible confounders, each unit rise in blood Pb was linked to a 7% increase in COPD risk (OR = 1.07, 95% CI: 1.00–1.14, *p* = 0.044). This finding reinforces the positive correlation between blood Pb levels and the risk of COPD. Quartile analysis further supported these results, showing that individuals in the highest quartile of blood Pb (Q4) exhibited a substantially greater risk of COPD compared to those in the lowest quartile (Q1) (OR = 1.87, 95% CI: 1.37–2.55, *P* for trend < 0.01) (Table 3).

**Table 3:** Multivariable logistic regression models for the association between blood Pb and COPD.

Variable	Model I (unadjusted)		Model II (demographics adjusted)		Model III (fully adjusted)	
	OR (95% CI)	*p*-value	OR (95% CI)	*p*-value	OR (95% CI)	*p*-value
Pb Continuous	1.11 (1.05, 1.17)	0.0002	1.05 (0.99, 1.12)	0.0981	1.07 (1.00, 1.14)	0.0445
Categories						
Quartile1	Ref		Ref		Ref	
Quartile2	1.71 (1.32, 2.23)	<0.0001	1.33 (1.01, 1.75)	0.0420	1.42 (1.06, 1.91)	0.0199
Quartile3	2.09 (1.62, 2.70)	<0.0001	1.40 (1.06, 1.85)	0.0170	1.54 (1.14, 2.07)	0.0046
Quartile4	2.60 (2.03, 3.33)	<0.0001	1.58 (1.19, 2.10)	0.0015	1.87 (1.37, 2.55)	<0.0001
*P* for trend	1.61 (1.42, 1.82)	<0.0001	1.23 (1.06, 1.42)	0.0063	1.34 (1.14, 1.58)	0.0004

OR, odds ratios; CI, confidence intervals; Ref, Reference. Model I, Unadjusted, without covariate adjustment; Model II, Adjusted for basic demographic factors, including age, sex, and race; Model III, Fully adjusted for demographic, behavioral, and health-related factors, including marital status, educational attainment, BMI, smoking habits, alcohol use, and comorbidities such as hypertension, diabetes, asthma, heart disease, and cancer.

In this study, heavy metal concentrations were examined both as continuous measures and by dividing them into quartiles as categorical variables. The results overall provide evidence supporting the hypothesis that elevated blood concentrations of heavy metals, especially Cd and Pb, are closely linked to a heightened risk of COPD.

### 3.3 Analysis of curve fitting

**Figure 2:**
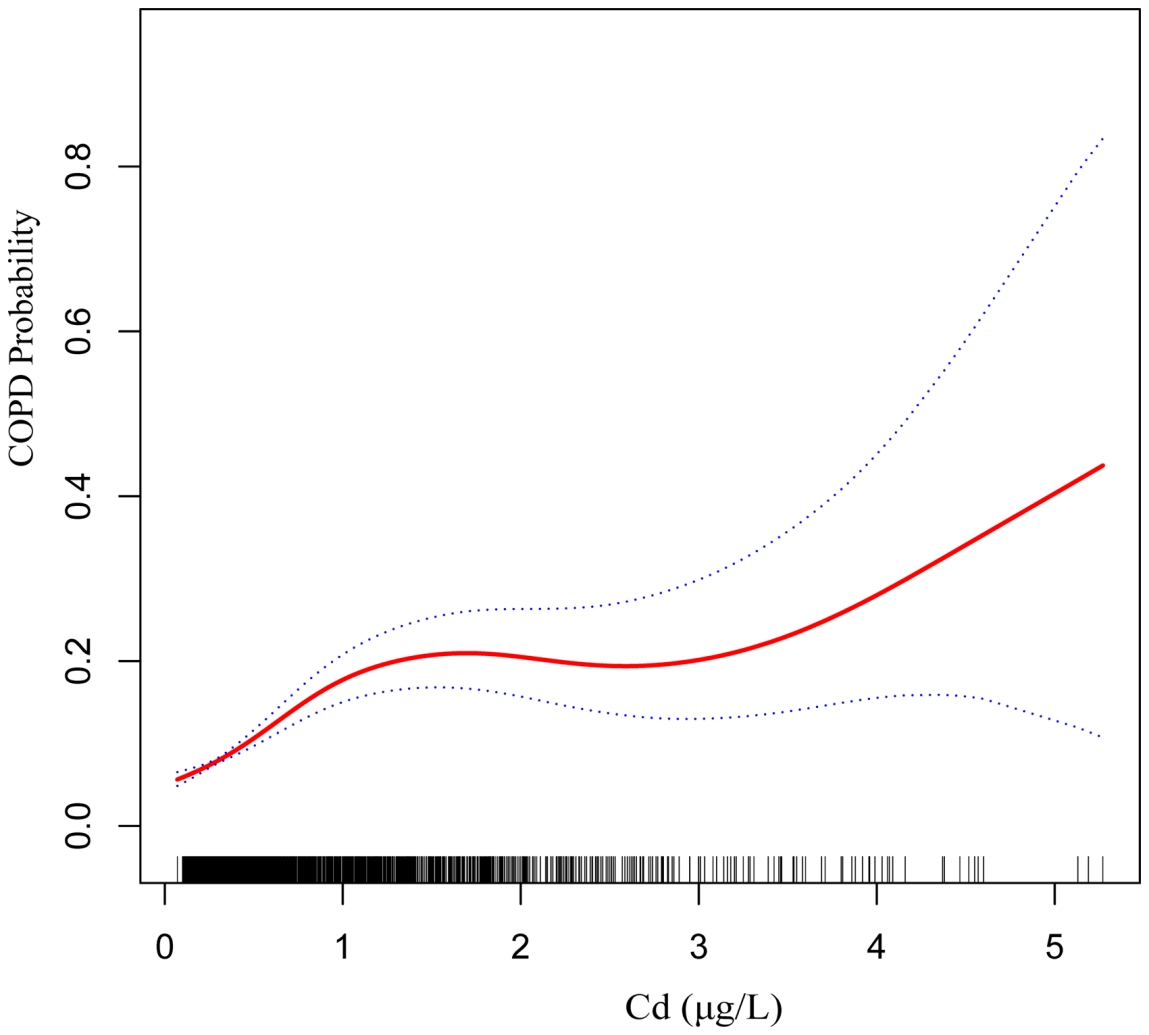
The smoothed curve between Cd and COPD is depicted in red, with blue bars representing the 95% CI.

**Figure 3:**
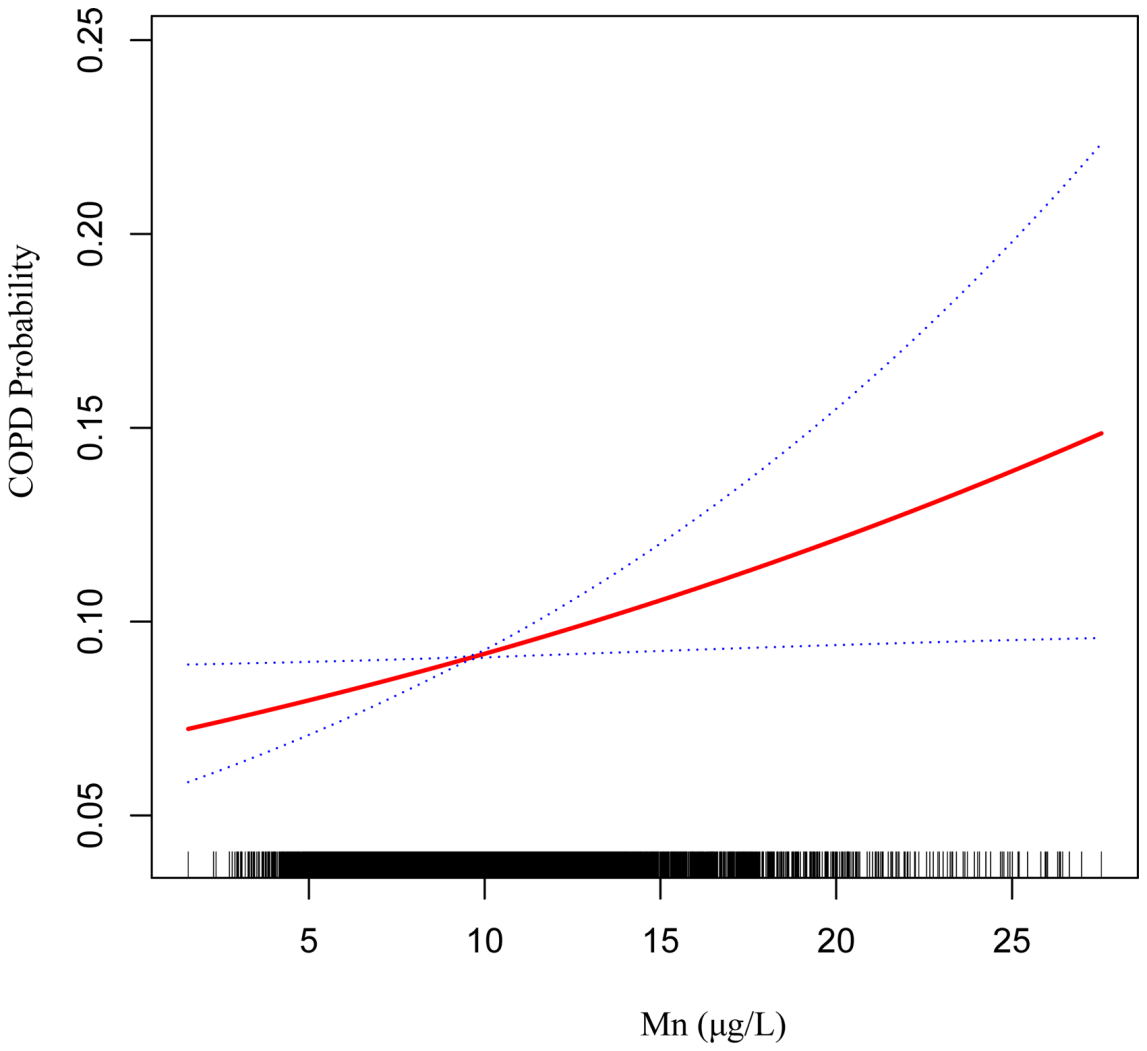
The smoothed curve between Mn and COPD is depicted in red, with blue bars representing the 95% CI.

The findings from Model III suggest a nonlinear positive relationship between blood concentrations of Cd and Mn and the occurrence of COPD (Figures 2, 3).

### 3.4 Subgroup analysis

**Figure 4:**
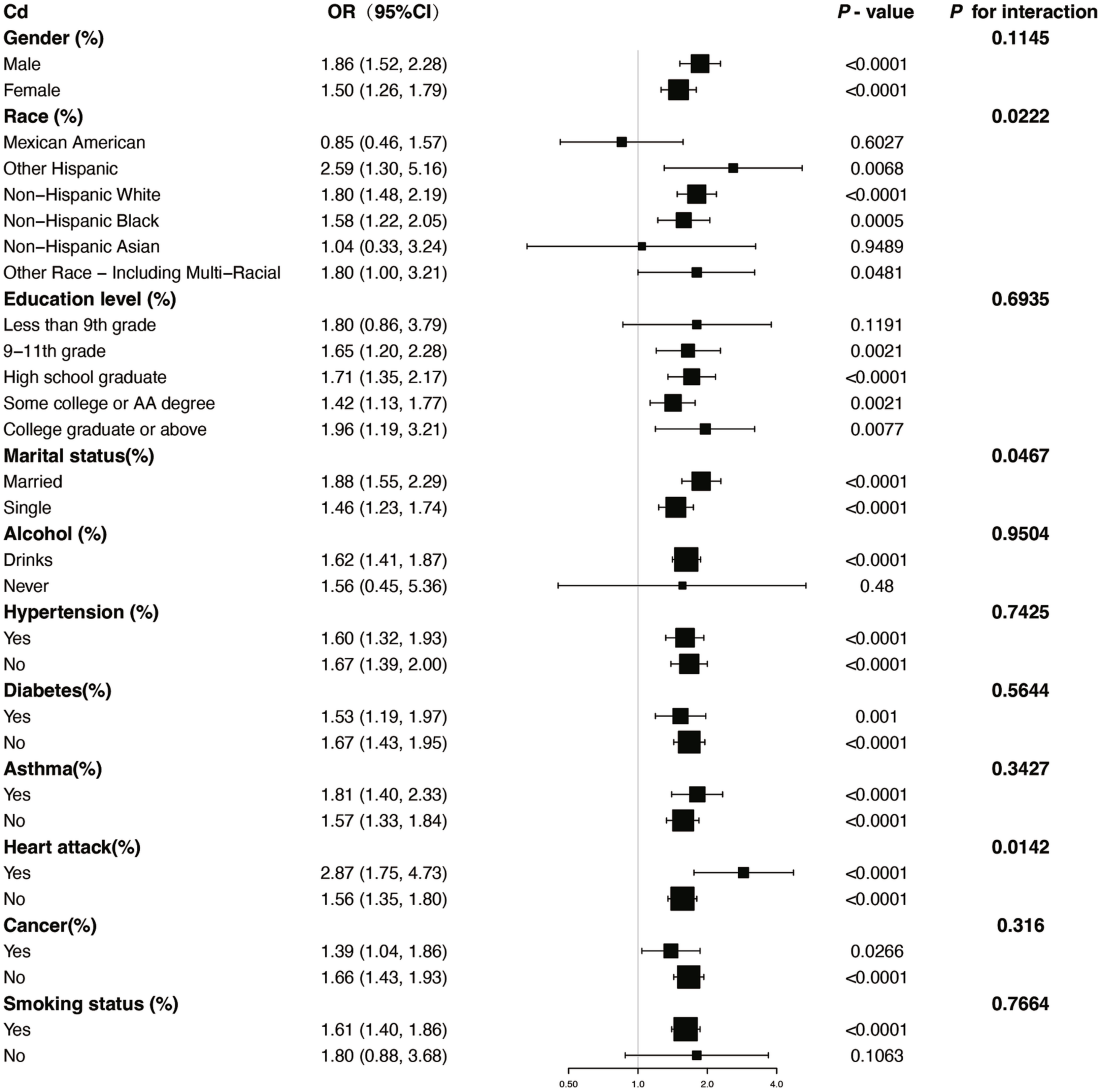
Subgroup stratified analysis of Cd and COPD with adjustments from Model III. OR, odds ratios; CI, confidence intervals.

**Figure 5:**
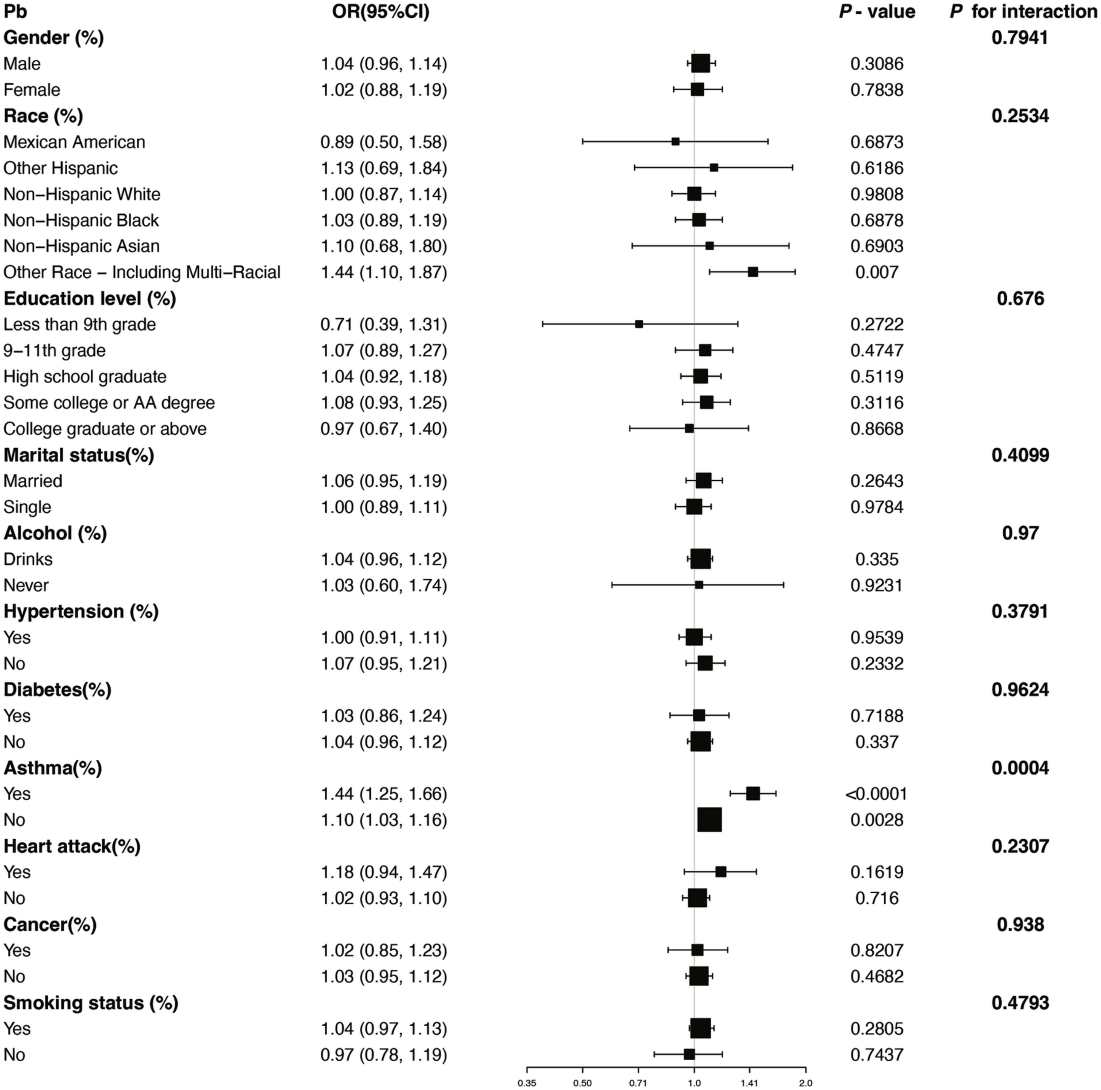
Subgroup stratified analysis of Pb and COPD with adjustments from Model III. OR, odds ratios; CI, confidence intervals.

We performed several subgroup analyses and interaction tests across various covariates to evaluate the strength of the relationship between blood concentrations of Pb and Cd and COPD prevalence, and to investigate potential variations among different population groups (Figures 4, 5). The results demonstrated a significant positive association between blood concentrations of Pb and Cd and COPD prevalence in most subgroups. Specifically, for Pb, a significant interaction was observed with asthma status (*P* for interaction = 0.0004). For Cd, there was a notable interaction with heart disease status (*P* for interaction = 0.0142). These results indicate that specific subgroup characteristics, including conditions like asthma and heart disease, could modify the association between blood heavy metal concentrations and the risk of COPD. Overall, while the association between Pb and Cd concentrations and COPD risk was consistent across most subgroups, the observed differences in specific populations warrant further investigation.

### 3.5 ROC analysis

**Figure 6:**
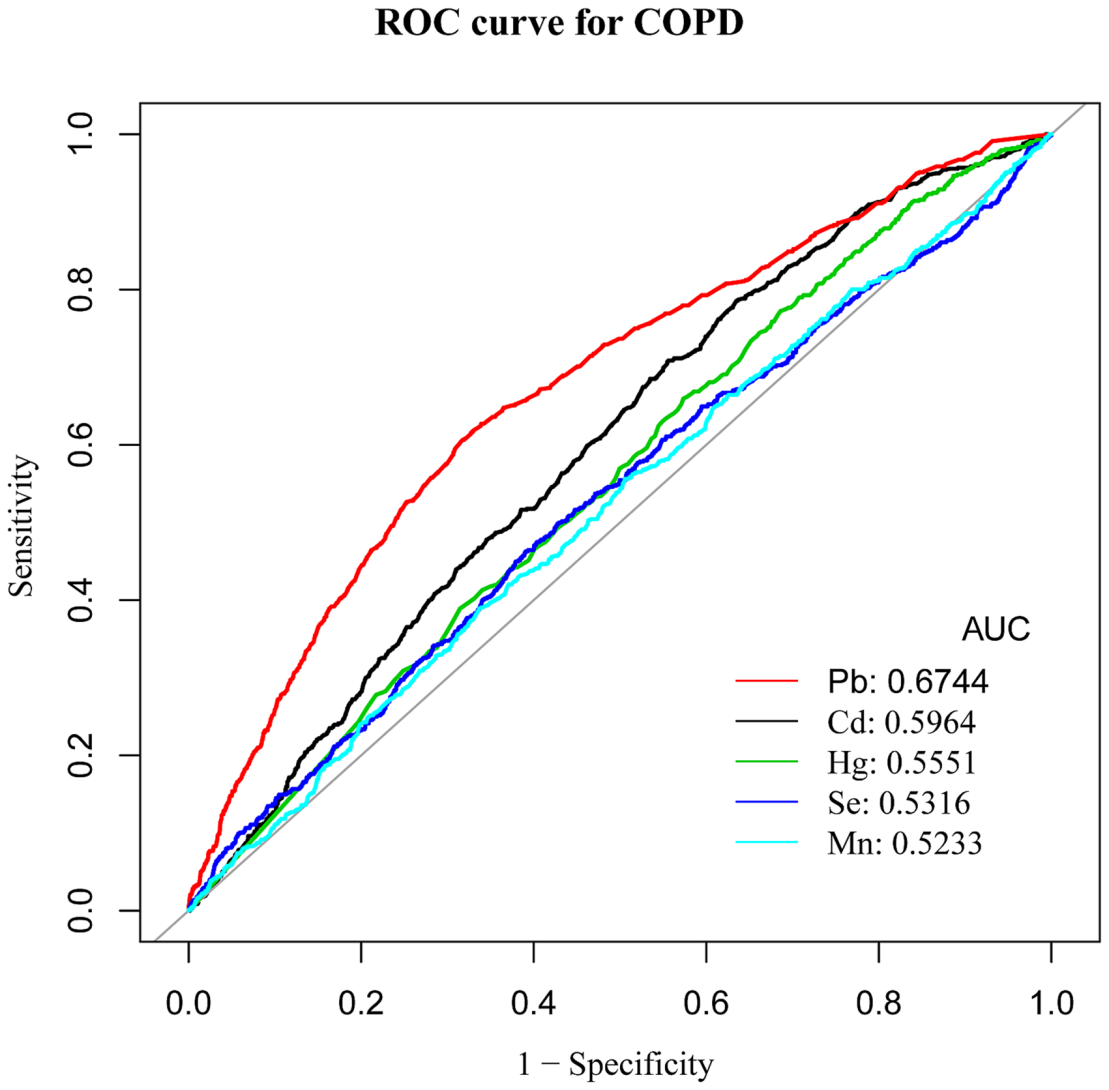
ROC curve for COPD. ROC, Receiver Operating Characteristic; AUC, Area Under the Curve.

Receiver operating characteristic (ROC) curve analysis was conducted to evaluate the discriminative ability of blood heavy metal concentrations in predicting COPD risk, with Area Under the Curve (AUC) values calculated for each metal. ROC analysis revealed that Pb had the highest predictive value for COPD among the metals studied (AUC = 0.6744, 95% CI: 0.6528–0.696), although the overall discriminative performance was modest. The AUCs for Cd, Hg, Se, and Mn were all below 0.6, indicating limited predictive utility. In comparison, the AUCs for Cd, Hg, Se, and Mn were 0.5964, 0.5551, 0.5316, and 0.5233, respectively. Although these AUC values were all above 0.5, suggesting some predictive potential, their performance was inferior to that of Pb (Table 4). Therefore, Pb was identified as the best predictor of COPD risk among the heavy metals examined in this study, indicating that blood Pb levels have greater diagnostic value in assessing COPD risk. These findings highlight the significant advantage of Pb as a predictor of COPD, outperforming other heavy metal indicators (Figure 6).

**Table 4:** Comparison of AUC values for different heavy metal levels.

Test	AUC	95%CI low	95%CI UPP	Best threshold	Specificity	Sensitivity
COPD						
Pb	0.6744	0.6528	0.696	0.4195	0.6824	0.6065
Cd	0.5964	0.5753	0.6176	0.7925	0.4438	0.7086
Hg	0.5551	0.5333	0.5768	1.155	0.3141	0.7722
Se	0.5316	0.5079	0.5553	177.595	0.6091	0.463
Mn	0.5233	0.5002	0.5464	7.965	0.6629	0.3891

AUC, Area Under the Curve; CI, confidence intervals.

## 4 Discussion

This research utilized data from NHANES to explore the relationship between blood levels of heavy metals—specifically Pb, Cd, Hg, Mn, and Se—and the prevalence of COPD. As a foundational step in environmental epidemiology, this large-scale, nationally representative cross-sectional study offers substantial strategic relevance. It serves a pivotal role in hypothesis generation by helping to screen multiple potential risk factors and identify high-priority targets—such as cadmium and lead in our study—that warrant investigation in future prospective studies. The results show a significant positive association between elevated blood concentrations of Pb and Cd and an increased risk of COPD, suggesting that heavy metal exposure may be linked to COPD risk. Further analysis revealed that higher blood Cd and Pb levels were associated with an increased risk of COPD even after adjusting for pertinent covariates, with the strongest associations observed in the highest exposure quartiles compared to the lowest. This significant trend across exposure quartiles supports their association with COPD risk, suggesting a potential etiological role as contributors to this disease.

Subgroup analysis further demonstrated significant interactions between heavy metal exposure and specific health conditions. For Pb, the association with COPD risk was significantly influenced by asthma status, suggesting that individuals with asthma may experience a heightened susceptibility to Pb-related respiratory effects. Similarly, for Cd, heart disease status showed a significant interaction, indicating that preexisting cardiovascular conditions may exacerbate the respiratory impacts of Cd exposure. Additionally, the nonlinear relationships observed between Cd and Mn levels and COPD risk suggest that the health impacts of these metals may vary depending on exposure levels, warranting further investigation into potential threshold effects.

ROC analysis identified Pb as the most accurate predictor of COPD risk among the studied heavy metals. While Pb showed the highest AUC value among the metals studied, its overall predictive performance was modest, indicating that its utility as a biomarker requires further validation. While other heavy metals demonstrated some predictive capability, their diagnostic utility appears limited in comparison. These results highlight the importance of prioritizing Pb in future risk assessment and intervention strategies.

This study provides evidence linking elevated blood levels of heavy metals to increased COPD risk, but further research is needed to clarify the underlying biological mechanisms. Understanding how specific heavy metals influence chronic inflammation, oxidative stress, and tissue repair may reveal novel therapeutic targets for preventing or mitigating COPD progression. Additionally, future studies should investigate the long-term effects of heavy metal exposure in prospective cohorts to establish causal relationships. Such research could also explore the differential impacts of heavy metals across diverse populations, considering socioeconomic and occupational factors that may contribute to exposure disparities. These insights could inform targeted public health interventions aimed at reducing heavy metal exposure and mitigating its health impacts.

As far as we are aware, this study is the first to investigate the link between blood levels of five specific heavy metals and COPD risk utilizing NHANES data from 2017 to 2020. Earlier research has examined the associations between three blood metals, six flavonoid compounds, total flavonoids, and COPD in individuals aged over 40 using previous NHANES datasets (24). Another study analyzed the relationship between copper exposure and COPD risk using NHANES data from 2015 to 2016 (28). Additionally, a recent longitudinal cohort study explored the association between Cd exposure and pulmonary function decline in COPD patients, highlighting a significant relationship mediated by oxidative stress over a two-year follow-up period (29). Our study, based on a larger and more recent dataset, investigates a broader range of blood metals and includes subgroup analyses, curve fitting, and comparative assessments of the predictive performance of different blood metals using ROC analysis.

The potential associations between these heavy metals and COPD likely involve multiple complex biological mechanisms. The pathogenesis of COPD itself is multifaceted, primarily involving chronic inflammation, protease-antiprotease imbalance, oxidative stress, apoptosis, and impaired tissue repair (30–33). Exposure to heavy metals, especially cadmium (Cd) and lead (Pb), can significantly contribute to these pathological processes (34, 35).

A primary shared mechanism is the induction of severe oxidative stress. Both heavy metals are known to disrupt cellular redox balance. Cd, often entering the body through inhalation of contaminated air or ingestion of polluted food (18), has been shown to directly increase the production of reactive oxygen species (ROS) while concurrently decreasing the activity of crucial antioxidant enzymes like superoxide dismutase (SOD) and glutathione peroxidase (GPx) (36–38). Similarly, Pb exposure inhibits these same antioxidant enzymes, leading to an accumulation of ROS and elevated cellular oxidative stress (21, 39). This shared mechanism of intense oxidative stress damages cellular components and DNA, ultimately promoting the apoptosis (cell death) of alveolar and bronchial epithelial cells (40).

Furthermore, both metals are potent inducers of pulmonary inflammation. Cd exposure has been reported to stimulate the release of pro-inflammatory cytokines, including TNF-*α* and IL-6, which aggravates lung tissue damage (41–43). Likewise, Pb, which tends to accumulate in organs like the lungs after exposure (44, 45), also elevates the levels of inflammatory cytokines (46). This cytokine release activates downstream signaling pathways that contribute to the chronic inflammation and airway remodeling characteristic of COPD.

In addition to Cd and Pb, other heavy metals, including Hg, Se, and Mn, have been suggested to be potentially linked to the development of COPD. Hg exposure can influence the progression of COPD by inducing oxidative stress and inflammatory responses. The toxic mechanisms of Hg primarily involve disrupting cellular redox balance and triggering abnormal immune reactions, which lead to lung inflammation and tissue damage (46–48). While Se and Mn are essential trace elements at normal levels, excessive exposure to these metals can induce oxidative stress and inflammation, further impairing respiratory health (49). These heavy metals may jointly play a role in the onset and progression of COPD through different biological pathways. Further studies are needed to clarify the precise mechanisms through which these metals affect COPD and to explore possible intervention strategies.

This study possesses several notable strengths. Firstly, it utilizes nationally representative data, which improves the generalizability of the results and enables analysis of the link between heavy metal exposure and COPD risk across diverse population groups. Secondly, the substantial sample size allows for a robust exploration of the associations between heavy metal levels and COPD within various subgroups. A large sample size not only increases the reliability of statistical analyses but also allows for more detailed subgroup analyses to reveal the differential effects of heavy metal exposure across different demographic characteristics. Additionally, we performed smoothing curve fitting analyses for Cd and Mn levels, uncovering potential nonlinear relationships between these metals and COPD risk. Furthermore, we conducted ROC analyses for all five heavy metals (Cd, Pb, Hg, Se, and Mn) to evaluate their sensitivity and specificity in predicting COPD risk. Nonetheless, this study has certain limitations. Primarily, the cross-sectional nature of the study design prevents the establishment of a causal link between heavy metal exposure and COPD and does not rule out the possibility of reverse causality. We can only identify associations, not determine whether heavy metal exposure directly causes COPD. Moreover, the diagnosis of COPD in this study was based on self-reported data from participants, without objective confirmation via spirometry. Although this method allows for efficient data collection from large populations, it is susceptible to recall bias and potential inaccuracies in the information provided. Such information bias could affect the accuracy of the findings, particularly when assessing COPD prevalence. Furthermore, to address potential confounding, we applied multivariable models with stepwise adjustments for demographic, behavioral, and health-related factors, alongside subgroup analyses to explore effect modifications across different population groups. These methods minimized the impact of known confounders and provided insights into potential differential risks. However, we recognize that residual confounding may still exist due to unmeasured or unobservable factors. Furthermore, a key limitation is that our study evaluated the association of each heavy metal individually and did not assess the potential synergistic or antagonistic effects of co-exposure to metal mixtures. Human exposure typically involves complex mixtures of environmental contaminants. Assessing potential interactions among these requires specialized statistical approaches, such as Weighted Quantile Sum regression and Bayesian Kernel Machine Regression. These considerations underscore the need for future studies employing longitudinal designs and objective diagnostic measures to better assess causal relationships and the health effects of complex metal mixtures.

In summary, this study provides nationally representative evidence linking elevated blood levels of cadmium and lead to a higher prevalence of COPD. These findings underscore the public health implications of environmental heavy metal exposure and highlight these metals as important environmental risk markers associated with COPD. Therefore, future research employing prospective cohort designs and objective spirometry measures is essential to confirm these associations and elucidate the underlying mechanisms.

## 5 Conclusion

This cross-sectional study provides compelling evidence of significant associations between specific blood heavy metal concentrations and COPD risk in a nationally representative sample. Our findings demonstrate that elevated blood levels of Cd and Pb are independently associated with increased COPD risk, even after adjusting for multiple confounding factors. The subgroup analyses showed that these associations remained consistent across most population subgroups, with specific interactions only observed between Pb and asthma status, and between Cd and heart disease status. The smoothing curve analyses revealed notable nonlinear positive associations between both Cd and Mn levels and COPD risk, while ROC curve analysis identified Pb as having the highest predictive capability among the five metals studied.

These results underscore the need for prospective cohort studies to establish causal relationships and further investigate the pathways through which heavy metals contribute to COPD development. Additionally, future research should focus on identifying high-risk populations and developing targeted intervention strategies to reduce heavy metal exposure and mitigate its health impacts.

## Data Availability

Publicly available datasets were analyzed in this study. This data can be found at: https://www.cdc.gov/nchs/nhanes/index.htm.

## Footnotes

^1^ www.cdc.gov/nchs/nhanes/
